# Cryovolcanism on the Earth: Origin of a Spectacular Crater in the Yamal Peninsula (Russia)

**DOI:** 10.1038/s41598-018-31858-9

**Published:** 2018-09-10

**Authors:** Sergey N. Buldovicz, Vanda Z. Khilimonyuk, Andrey Y. Bychkov, Evgeny N. Ospennikov, Sergey A. Vorobyev, Aleksey Y. Gunar, Evgeny I. Gorshkov, Evgeny M. Chuvilin, Maria Y. Cherbunina, Pavel I. Kotov, Natalia V. Lubnina, Rimma G. Motenko, Ruslan M. Amanzhurov

**Affiliations:** Lomonosov Moscow State University, Leninskie Gory, 1, Moscow, 119992 Russia

## Abstract

Geological activity on icy planets and planetoids includes cryovolcanism. Until recently, most research on terrestrial permafrost has been engineering-oriented, and many related phenomena have received too little attention. Although fast processes in the Earth’s cryosphere were known before, they have never been attributed to cryovolcanism. The discovery of a couple of tens of meters wide crater in the Yamal Peninsula aroused numerous hypotheses of its origin, including a meteorite impact or migration of deep gas as a result of global warming. However, the origin of the Yamal crater can be explained in terms of cryospheric processes. Thus, the Yamal crater appears to result from collapse of a large pingo, which formed within a thaw lake when it shoaled and dried out allowing a large talik (that is layer or body of unfrozen ground in a permafrost area) below it to freeze back. The pingo collapsed under cryogenic hydrostatic pressure built up in the closed system of the freezing talik. This happened before the freezing completed, when a core of wet ground remained unfrozen and stored a huge amount of carbon dioxide dissolved in pore water. This eventually reached gas-phase saturation, and the resulting overpressure came to exceed the lithospheric confining stress and the strength of the overlying ice. As the pingo exploded, the demarcation of the crater followed the cylindrical shape of the remnant talik core.

## Introduction

Volcanism shaped the surface of some larger rocky bodies in the Solar System, but currently it is restricted to the Earth and Io. Cryovolcanism remains an important form of geological activity on icy planets and planetoids^1–4^. Traditionally, most studies of terrestrial permafrost were engineering-oriented, and many related cryogenic phenomena received little attention. Although fast processes in the Earth’s cryosphere were known before, they have until now never been attributed to cryovolcanism. The recent discovery of a couple of tens of meters wide crater in the Yamal Peninsula aroused numerous hypotheses of its origin, including a meteorite impact or migration of deep gas as a result of global warming^5,6^. On the other hand, explosions of cryogenic structures are well known although rare^7,8^. But in 2017 there was a report about two similar, but smaller, explosions on the Yamal peninsula^9^. Perhaps the impact of global warming can provoke such explosions, as the depth of thaw increases and the frozen cap is easily destroyed. Here we argue thatthe origin of the extraordinary large Yamal crater can be also explained in terms of these explosive cryospheric processes.

**Figure 1:**
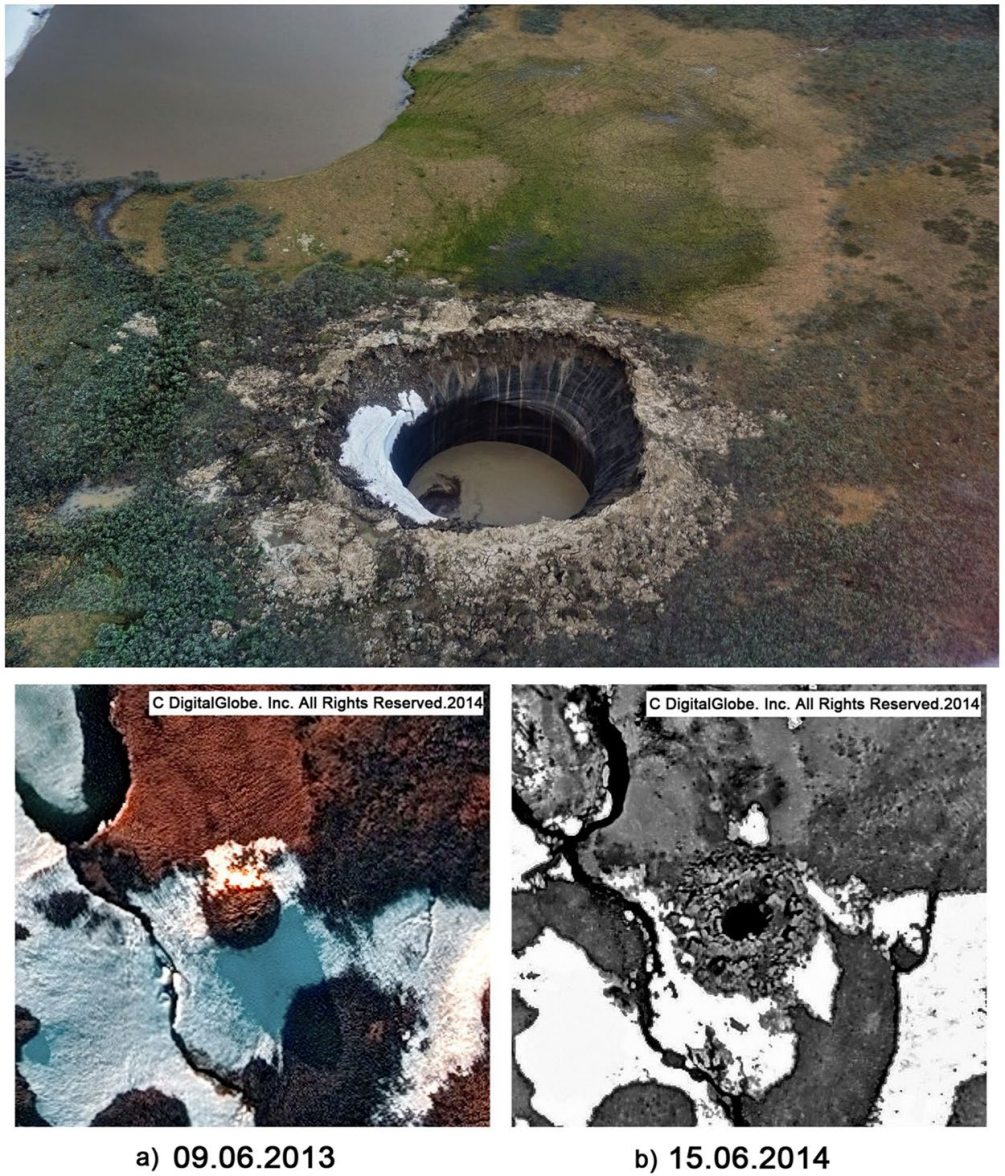
Yamal crater on July 15, 2015 (Photograph by Ruslan Amanzhurov) and high-resolution satellite imagery of 2012 (**a**) and 2013 (**b**).

The crater was found in the summer of 2014 in the central Yamal Peninsula at N69.970965 E68.369575, 30 km southeast of the Bovanenkovo gas field. The crater is expressed as a hole, 20 m in diameter, with vertical walls in the lower half and an opening conical shape in the upper half, encircled with a parapet-like ridge of ejected material. Its depth exceeded 50 m with a maximum observed depth of 52 m measured on 16 July 2014^5,6,10^. The open void quickly filled with water (Fig. 1) and turned the crater into a lake in the autumn of 2016.

We visited the crater in June of 2015. It was then noticeable as an irregular circle, about 25 meters in diameter (Fig. S1), with eccentricity no more than 1 m. The water table of the lake was 18 meters below the ground surface. The approximately 5–10 m wide parapet of ejecta around the crater was 0.5 m to 3.5 m high, obviously with a volume inferior to that of the crater.

**Figure 2:**
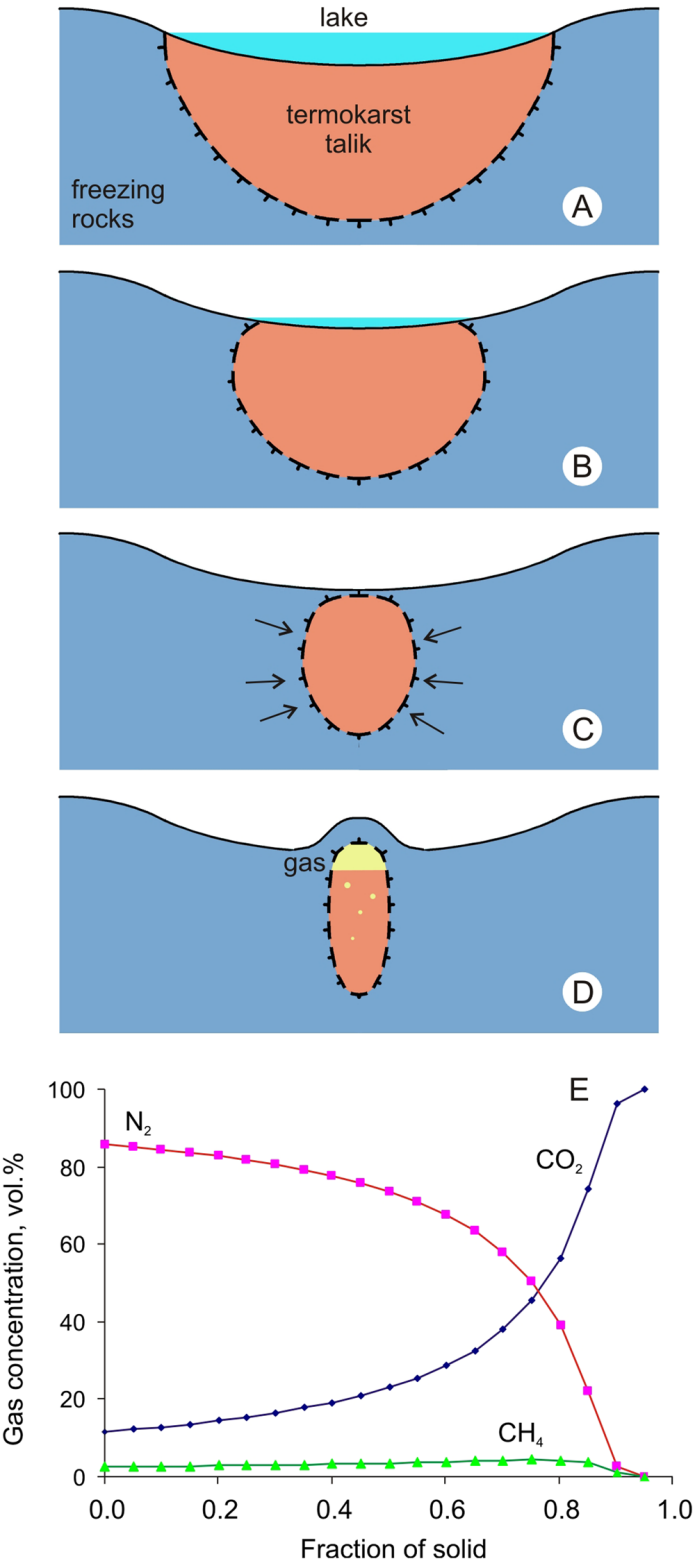
Freezing of the sub-lake talik and the formation of a pingo: (**A**) closed talik under the lake; (**B**) lateral freezing of the closed talik upon lake shrinking; (**C**) formation of a freezing closed talik; (**D**) growth of frost mound (pingo); (**E**) evolution of gas composition with freezing fraction increasing.

The Yamal crater is located in a zone of ice-rich continuous permafrost of −1 °C to −5 °C mean annual temperatures, with a bulk ice content of 30–65 vol %, although frequently confined to thick lenses of ground ice. The crater formed in a thermal-erosion valley incised into what has been described as marine terrace III^11^. Before 2013, there existed a large pingo in the place of the crater, 8 m high and with a base diameter of 50–55 m, according to high-resolution satellite imagery of 2012 and 2013 (Fig. 2).

Samples from boreholes along a profile that traversed the Yamal crater (Fig. S2) show large variations of cryostructures both laterally and with depth (Fig. S3). Quaternary sediments are either marine terrace or lake deposits. The cryostructures of marine terrace III (mIII_2−3_) deposits documented in borehole 1 are layered and lenticular-layered in the upper part and irregular reticulate at greater depths (Figs S4 and S5). Lake sediments (l_1_IV) and sediments subjected to repeated freezing (l_2_IV) show diverse cryostructures: lenticular, layered, suspended, irregular reticulate and massive (Figs S6 and S7).

Borehole 7, 17 m deep, located five meters north of the crater, penetrated ground ice 5.8 m below the surface, but failed to reach its base. It is columnar ice with alternated nearly vertical (80°) layers of bubbly clear and colored ice; the latter is stained with humic acid and hosts mineral inclusions (Figs S8–S10). The columnar ice is cut by milky-white intrusive ice of a younger generation. The crater walls expose complex relations among different types of ice (Fig. S7). Cryogenic structure indicates freezing propagation towards the crater.

We analyzed separately the compositions of mineral and ice (water) components in the collected permafrost samples. The element patterns correspond to two chemically different permafrost types (Figs S12 and S13). One is frozen marine terrace deposits (borehole 1 and upper section), which remained unaffected by thermokarst. Its ice component contains minor amounts of metals and organic carbon. Ice in permafrost of the other type contains up to 3.5 g/l dissolved organic carbon and melts to a dark brown alkaline (pH 8–9.5) solution. The contents of metals are low in the mineral component of sediments (except for SiO_2_, CaO, Na_2_O) but high in the ice. This distribution of elements may result from prolonged interaction of ground and melt water, which produced organic-metal compounds. The chemical interface appears to trace the upper boundary of a closed sub-lake talik.

Frozen sediments and ice of the study area are surprisingly rich in gases reaching 20 vol.%. The gaseous component (see Fig. S13 for its distribution) in sediments near the crater differs markedly from natural gas of the Bovanenkovo field in the concentrations of carbon dioxide, nitrogen, hydrogen, and methane homologs^12^. The carbon isotope composition of methane is typical of biogenic hydrocarbons (δ^13^C = −76‰ PDB). This difference disproves the hypothesis that the gas in the crater would come from deep Bovanenkovo reservoirs^6^. Prevalence of high normal alkanes (above C_19_H_40_) indicates that the hydrocarbons are derived from buried plant remnants.

Mathematical modeling was undertaken to study the changes in the boundary conditions on the surface that can produce a cylindrical, residual talik after long-term freezing of the primary talik (Fig. S1). Heat flow and freezing dynamics were calculated using the WARM software^13^.

Modeling was performed for different initial conditions. The results show that a talik in the shape of a vertical cylinder can form at the following prerequisites (Fig. 2).

1. A deep talik can form under quite a large long-lasting thermokarst (thaw) lake, about the size of the dry lake that accommodates the Yamal crater (400–500 m). According to our calculations, the formation of a 60–70 m deep talik requires about 3000 years (Fig. 2A).
2. Judging by the shape of the talik core and the thickness of frozen sediments above it (6–8 m), it formed by lateral freezing while the lake was shrinking (Fig. 2B).
3. The formation of a thin frozen cap over the talik core is possible in the conditions of a small shallow (slightly above a critical depth) dying lake and subzero mean annual temperatures of lake sediments. In this case, hydrostatic pressure can increase by water expulsed from permafrost in a closed system, which produced a pingo for the past 100 years (Fig. 2C,D).

With the 2.2·10^5^ m^3^ volume of frozen ground (the former talik) and an average gas content of 14 vol.%, the volume of gas at normal conditions can be estimated at ~3·10^4^ m^3^. As the talik was freezing back, a substantial portion of gas may have been expulsed inward to sediments remaining unfrozen, but the exact amount of gas in the unfrozen talik core is unknown. It may largely consist of carbon dioxide, which is highly soluble in water (Fig. 2E). The volume of gas in the core, under normal conditions, may at most correspond to all its space. However, the pressurized dissolved gas obviously occupies a smaller volume, and the energy of gas expansion may be the main driving force of crater formation. Gas pressure in the closed talik produced a mound (pingo or bulgunnyakh) on the ground surface.

Thus, the Yamal crater appears to result from collapse of a large pingo, which formed within a thaw lake when it shoaled and dried out allowing a large talik below it to freeze back. The pingo collapsed under cryogenic hydrostatic pressure built-up in the closed system of the freezing talik. This happened before the freezing completed, when a core of wet ground remained unfrozen and stored a huge amount of carbon dioxide dissolved in pore water. As the pingo exploded, the demarcation of the resulting crater followed the cylindrical shape of the remnant talik core. Such formis unusual for an ordinary talik.

At a low temperature on the talik surface, and in the presence of a long-lasting pingo, the frozen cap turned out to be too thick to break down under hydrostatic pressure. Our calculations show that the equilibrium pressure of the talik core under a 6−8 m thick cap can reach 5 bar, while about 10 bar is required for the breakdown of such a cap. This pressure is close to the invariant point in the H_2_O−CO_2_ system (P = 9.82 bar; T = −1.4 °C) at which liquid water can coexist with ice, carbon dioxide clathrate and gaseous carbon dioxide^14–16^. The formation of carbon dioxide clathrate is especially possible at the bottom of the talik core where pressure may reach 15 bar. This mechanism works only in gas-saturated systems. Destruction of the pingo with a small amount of gas leads to a liquid water ejection, but not to an explosion.

The talik core had a cylindrical (tube) shape with its diameter corresponding to the original crater size (17 m) and was 60 m deep, or slightly deeper than the crater. Its filling had a complex vertical structure: mainly thaw soil containing carbon dioxide hydrates below, liquid water with dissolved gas (mainly carbon dioxide) in the middle, and mainly gas on the top. Freezing from above produced porous ice with abundant carbon dioxide gas bubbles under a pressure of <10 bar. This ice was described in early studies of the crater^7^. The final freezing stage is sketched in Fig. 2.

**Figure 3:**
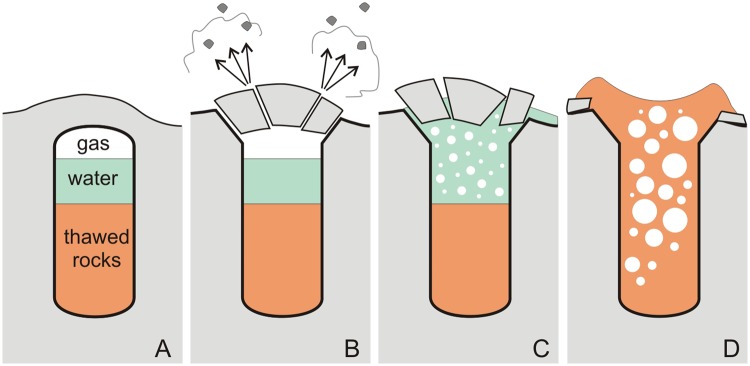
Pre-explosion (**A**), pneumatic (**B**), hydraulic (**C**), and phreatic (**D**) stages of a cryogenic eruption.

Cryogenic eruption was triggered by thermal contraction cracks (ice wedges) in the frozen cap and comprised several stages (Fig. 3).

1. Pneumatic stage (few minutes) of gassing from the talik top: gas pressurized to ~10 bar releases through cracks and expands adiabatically. Initial maximum gas temperature was −1.4 °C and decreases as a result of the adiabatic expansion of carbon dioxide. The cold gas jets affected shrubs growing near the cracks while pieces of dry ice became dispersed around the eruption center. The energy of the gas breaking through cracks was sufficient to disperse pieces of ground for a long distance.
2. Hydraulic stage (few hours) of water outpouring from the crater: pressure drop leads to CO_2_ degassing from water and ascent of the gas-water mixture (“champagne” effect). The mixture also contained some ice produced by partial freezing of water. The rising ice-water-gas mixture broke the permafrost cap and threw it out from the crater. A parapet began forming around the crater.
3. Phreatic stage (5–25 hours) of unfrozen soil eruption: gas releases either from pore water in unfrozen soil or from decomposed carbon dioxide hydrates. The decomposition of gas hydrates is relatively slow, and is further decelerated by freezing. However, the process releases a large amount of gas, which pushes unfrozen soil upward along the chimney producing a symmetrical ridge around the crater. While rising, unfrozen soil partially freezes back and forms lumps preserved around the crater. The phreatic stage is the longest because gas hydrate decomposition is slow. It may last several days, or as long as the degassing is able to maintain eruption.

Cryovolcanism has been commonly assumed to have a warm source at the depth^9^. This is partly true, but our results show that eruption may result from water crystallization rather than warming. The water-to-ice transition increases pressure and triggers a catastrophic explosion. The mechanism proposed for the Yamal crater may explain the formation of water jets on Saturn’s moon Enceladus^17^. Injection of liquid water into the icy crust would produce water diapirs pressurized upon freezing. Quite strong explosions can occur under these conditions.

The development of the Arctic leads to discovery of previously unknown traces of cryogenic phenomena such as the Yamal crater. Our observations show that processes in the Earth’s cryosphere can be very fast and produce short-lived objects. Therefore, they were rarely detected prior to the current active development of Arctic areas. There may exist other types of cryovolcanism besides the reported case, and phenomena more perilous for human infrastructure, or even the climate, may arise in the future.

## Materials and Methods

For the separate analysis of the mineral matrix of ground and ice a cut core was placed in plastic bags and thawed at ambient temperatures (8–15 °C). Water sampling occurred within one day. At high ice content of the sample, the melted water was trapped by ICP−MS protocol. The solution was filtered through a membrane filter “Vladipor” with a pore size of 0.45 microns using a syringe nozzle. 15 ml of water were collected in a plastic vessel and immediately were conserved by high purity HNO_3_ (Merck) to a concentration of 3%. Simultaneously with the sampling, the following parameters of the solution were determined: electrical conductivity using a conductivity meter Expert 002 and potentiometric measurement of pH, Eh and the activity of fluoride ions. Potentiometric measurements were conducted with the pH-meter Expert-001 and ion-selective electrodes silver chloride reference electrode EVL-1M3.1. A glass electrode ECL-43–07 was used to determine the pH. The redox potential was determined using two electrodes - glass redox EO-01 and a platinum EVP-1. The combination of these electrodes allows to determine the parameters of potential-reaction.

For studies of the gas composition a standard volume of core (400 cm^3^) was placed in a sterile glass container with 100 cm^3^ of saturated sodium chloride solution. The gas chromatographic analysis was conducted on a Crystal-2000M chromatograph (Chromatec, Russia).

In the laboratory, samples were dried in an oven at 105 °C and crushed in a porcelain mortar. From the crushed sample a representative portion of 5 g was selected, which was grind to an analytic powder. The content of macro components was determined by X-ray fluorescence (PW 2400, Philips Analytical™, trace elements – ICP-MS by Element-2, Thermo Fisher Scientific™.

## Electronic supplementary material

Supplementary Materials

## References

[1] Roth L (2014). Transient water vapor at Europa’s South Pole. Science.

[2] Saur J (2011). Hubble space telescope/advanced camera for surveys observations of Europa’s atmospheric ultraviolet emission at Eastern Elongation. Astrophys. J..

[3] Soare RJ (2013). Possible crater-based pingos, paleolakes and periglacial landscapes at the high latitudes of Utopia Planitia, Mars. Icarus.

[4] Soare RJ, Conway SJ, Dohm JM, El-Maarry MR (2014). Possible open-system (hydraulic) pingos in and around the Argyre impact region of Mars. Earth and Planetary Science Letters.

[5] Leibman MO, Kizyakov AI, Plekhanov AV, Streletskaya ID (2014). New permafrost feature-deep crater in central Yamal (West Siberia, Russia) as a response to local climate fluctuations. Geography, Environment, Sustainability.

[6] Olenchenko VV (2015). Results of geophysical surveys of the area of “Yamal crater”, the new geological structure. Kriosfera Zemli.

[7] Mackay JR (1979). Pingos of the Tuktoyaktuk Peninsula Area, Northwest Territories. Géogr. Phys. Quat..

[8] Mackay JR (1998). Pingo growth and collapse, Tuktoyaktuk Peninsula Area, Western Arctic Coast, Canada: along-term field study. Géogr. Phys. Quat..

[9] Geissler, P. Cryovolcanism in the outer solar system. *The Encyclopedia of Volcanoes (Second Edition)*, 763–776 (2015).

[10] Kizyakov AI (2015). Geomorphological conditions of the gas-emission crater and its dynamics in Central Yamal. Kriosfera Zemli.

[11] Badu YB (2015). Ice content of cryogenic strata (permafrost interval) in gas-bearing structures, Northern Yamal. Earth’s Cryosphere.

[12] Bondarev VL (2008). Geochemical characteristics of the under Cenomanian deposits of the Yamal Peninsula (on the example of the Bovanenkovo oil and gas condensate field). Geologiya, geofizika i razrabotka neftyanykh i gazovykh mestorozhdeniy.

[13] Khrustalev, L. N., Emelyanov, N. V., Pustovoyt, G. P. & Yakovlev, S. V. The program for calculating the thermal interaction of engineering structures with permafrost WARM. *The certificate No. 940281 RosAPO* (1994).

[14] Fray N, Marboeuf U, Brissaud O, Schmitt B (2010). Equilibrium data of methane, carbon dioxide, and xenon clathrate hydrates below the freezing point of water. Applications to astrophysical environments. J. Chem. Eng. Data.

[15] Longhi J (2005). Phase equilibrium in the system CO_2_−H_2_O I: New equilibrium relations at low temperatures. Geochim. Cosmochim. Acta.

[16] Longhi J (2006). Phase equilibrium in the system CO_2_-H_2_O: Application to Mars. J. Geophys. Res..

[17] Waite JH (2009). Liquid water on Enceladus from observations of ammonia and ^40^Ar in the plume. Nature.

