# Supplementary material for: Cryovolcanism on the Earth: Origin of a Spectacular Crater in the Yamal Peninsula (Russia)
Source: Sci Rep. 2018 Sep 10;8:13534. doi: 10.1038/s41598-018-31858-9 (PMC6131154; doi:10.1038/s41598-018-31858-9)
Supplement: Supplementary file 1 — Supplementary Materials [file 41598_2018_31858_MOESM1_ESM.doc]

Supplementary Materials for

Cryovolcanism on the Earth: the Origin of the Spectacular Crater on Yamal Peninsula (Russia)

S. N. Buldovicz, V. Z. Khilimonyuk, A. Y. Bychkov, E. N. Ospennikov, S. A. Vorobyev, А. Y. Gunar, E. I. Gorshkov, E. M. Chuvilin, M. Y. Cherbunina, P. I. Kotov, N. V. Lubnina, R. G. Motenko, R. M. Amanzhurov

Correspondence to: andrewbychkov@rambler.ru

**This PDF file includes:**

SupplementaryText

Figs. S1 to S13

**Mathematical modeling of long-term evolution of sub-lake talik.**

Mathematical modeling was applied to determine the specifics and dynamics of change in upper bound conditions of soil surface, which in the course of long-term freezing of the primary talik can lead to formation of residual talik of mentioned shape.

Modeling of processes of heat transfer in the soils was calculated using software developed at the Department of geocryology, Faculty of geology, Lomonosov Moscow State University *(12)*. The program implements a solution for non-stationary heat conduction problems with arbitrary boundary conditions with the presence of moving phase boundaries in the reference zone.

After calculations of a series of thermal conditions the results showed that specific shape of talik can be formed during cryogenic transformation of initial mass of thawed soils according to different scenarios of change in upper boundary conditions, and thus the problem appears to be unsolvable in a retrospective way.

However, there are a number of conditions required to form the observed configuration of residual talik.

1. In order for talik to form at such depth, thickness of initial thaw soils formation has to be more than the depth that talik is found in its final stage of existence. Such thaw bulb can be formed only within the free water columnof a lake of sufficient size and life span. The size of the ancient lake hollow is 400-500 m, which fits these requirements quite well. A bit more strict condition is the life span of the lake: it has to be no less than 3000 years in order to form a talik of such thickness.

2. The formation of such "narrow" talik clearly involves its active lateral (subhorizontal) freezing as the result of size reduction of lake water area, gradual or abrupt.

3. At the final stage of pingo formation, prior to its destruction, natural conditions on the soil surface are imposed by special requirements.

The pingo growth starts only after complete isolation of talik area from the top with the beginning of long-term freezing of soils over its whole surface. Pingo is formed by reducing the density of water (thermal expansion) during its crystallization.

In order for such landform to be formed (with above-ground part volume of 8000 m^3^) , it is required to freeze 10 times more volume of water than its terrestrial part volume, and respectively the volume of thaw soils containing this water should be 2-3 times more (about 160000 – 200000 m^3^). It is clear that freezing process of such a volume of water-saturated thaw soils takes a long period of time.

On the other hand, thickness of the frozen overburden of the pingo before its destruction was about 6-9 m. Therefore, to form large pingo with thaw soils making its core and relatively thin frozen overburden, it is necessary for the freezing process to prevail in lateral directions than from above on the final stage of freezing of initial sub-lake talik. In this case, such combination is only possible if a small shallow overgrown residual pond, with negative, but close to 0° С average annual temperature of bottom sediments, existed for a long time; while temperature of surrounding surface area of the previously dried lake should also stay relatively low. All the above conditions can be met only in very rare environments, so this makes the distribution of the objects like Yamal crater to be uniqueIn this case, geocryologic conditions on the bottom of a single, round-shaped lake were observed. A two-dimensional radial system with axial symmetry of vertical center axis was calculated. The calculated area is a cylinder, on which a contour of a round lake is located in the middle of the circle surface , with a variable radius length that can be arbitrary changed with time.

Within the contour of the lake the temperature of the bottom sediments is set to +2° C, for the rest of the upper surface of the calculated area it is set to the average annual temperature of surrounding permafrost which is -4° C. The cylindrical calculated area with radius of 400 m along the lateral border is isolated. At the lower boundary of the area (500 m depth) a constant flow of heat from the depths is set (0.046 W/m^2^).

Thermal properties of the soils are the following: the heat conductivity in unfrozen and frozen state is respectively $\lambda_{th}$=1.74, $\lambda_{f}$=2.32 W/(m∙K); the volumetric heat capacity $C_{th}$=700, $C_{f}$=580 (W∙h)/(m^3^∙K); the volumetric heat of phase transition of water $Q_{ph}$=37100 (W∙h)/m^3^. These settings correspond with 40% volumetric iciness.

In these conditions, the thickness of permafrost at some distance from the lake is 200 m (*M* = 200 m), the initial radius (*R_l_*) of the lake is 120m (which is about half the radius of the lake hollow), and the thickness of stationary closed talik (*l_t_*) in this case is 100m.

Simulation was performed for different retrospective scenarios of the lake configuration. All of these scenarios in the final stage of talik degradation led to the formation of a cylindrical thawed zone with the configuration very close to that observed in nature. The following are the simulation results for one of the scenarios;

In the first stage the stationary temperature mode of the soils and thaw bulb configuration were reached. Then the change of geocryological situation during lake draining is observed.

In this scenario, at a certain time the initial radius of the lake, equal to 120m, begins to shrink with a constant speed of 5 cm/year (10 m in 200 years), but the surface temperature of the bottom sediments and surrounding surface remains unchanged. After reduction of the lake radius up to 40 m over 1600 years, freezing of talik starts from the top with the bottom sediments temperature about -0.2 – -0.3° С and continues for 300 years. All the changes in geocryological situation are shown in Figure 1 (blue colour for the frozen soils, yellow for the thawed). The residual thawed zone in the pingo core has a regular cylindrical shape with a radius of 10 m and 70 m vertical depth; the thickness of the frozen ground overburden is 6–8 m.


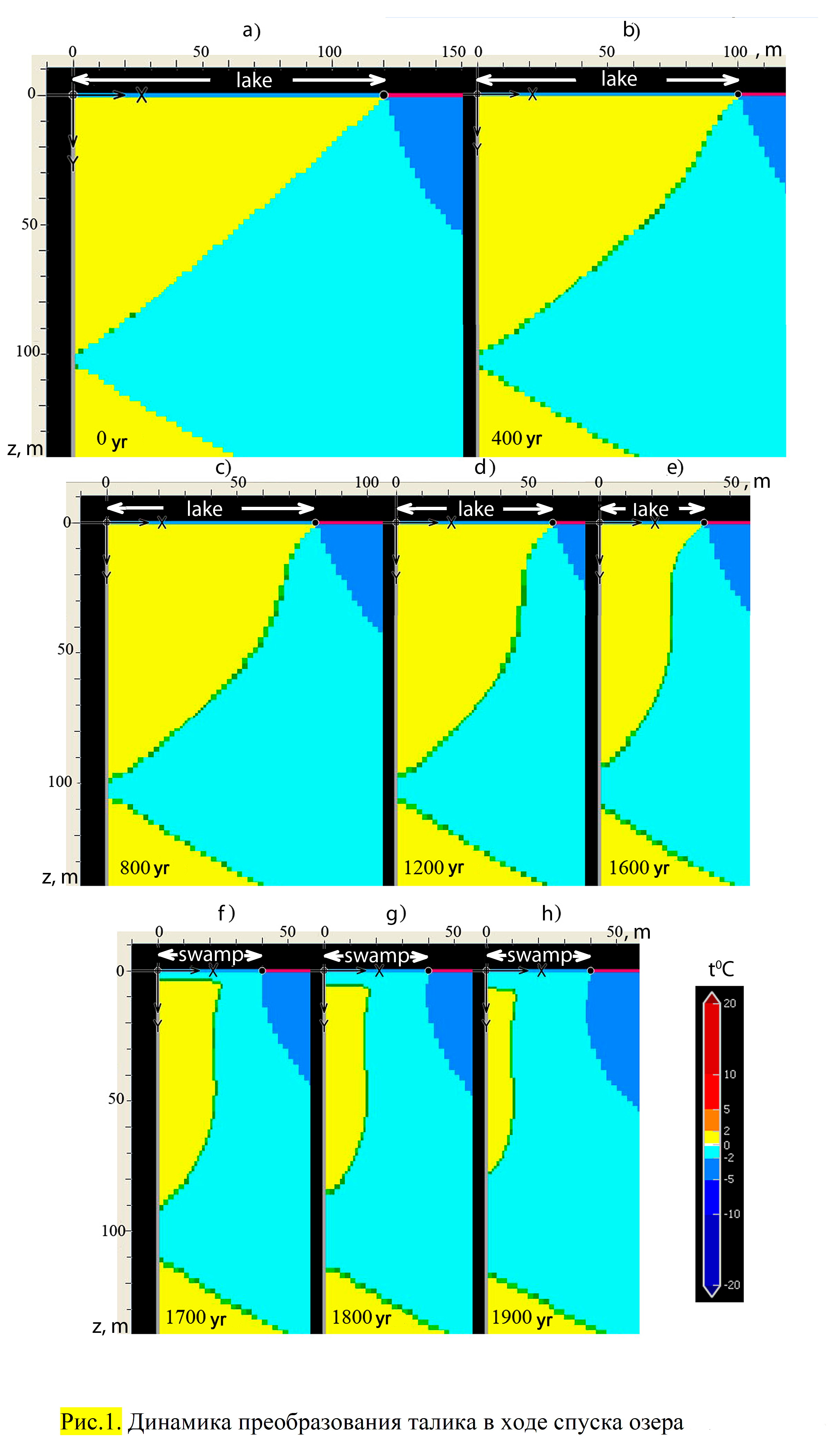


Fig S1. The talik dynamics during the lake draining (mathematical model).

Fig. S2. Topographic Scheme of the Crater Location.

Studied boreholes and profile are showed by blue line. Map was generated from a topographic survey by Ruslan Amanzhurov using ArcGis v.10.3 (license agreement 2009A3324).

Fig. S3. Cryogenic Structure of the Section.

1 - peat; 2 - loam; 3 - sandy loam; 4 - silt; 5 - clay; 6 - fine sand; 7 - intrusive ice; 8 - ice-rich ground; 9 - peat inclusion; 10 - turf inclusion; 11 - pebble inclusion; 12 - ferrugination traces. Section was generated using AutoCAD 2016 release.


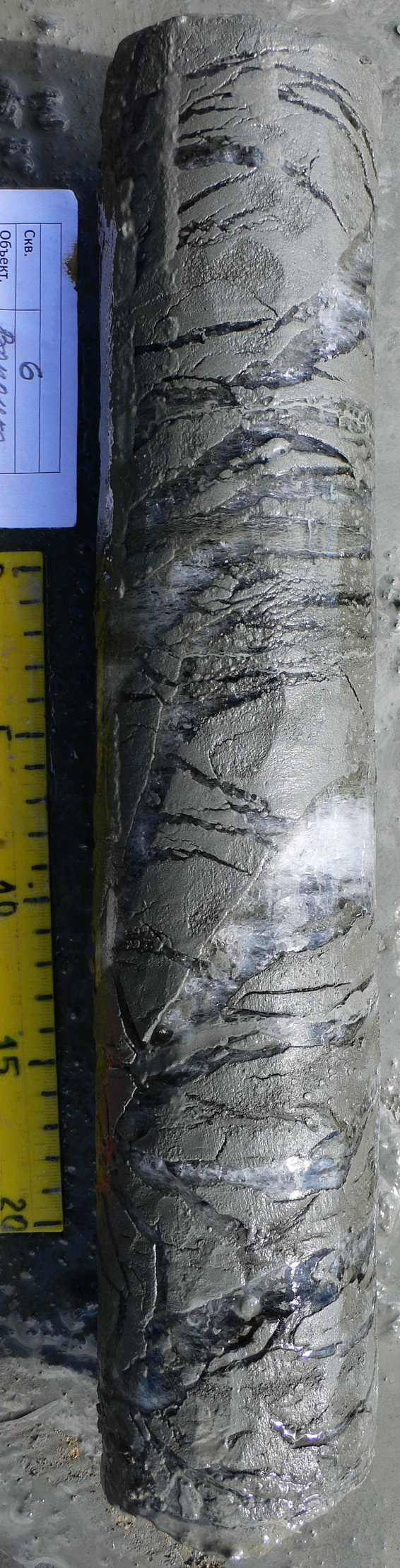


Fig. S4. Irregular reticulate cryostrucure.

Borehole 1, 8.5-8.75 m depth. Centimeter ruler for scale. Photograph by Vanda Khilimonyuk.

A


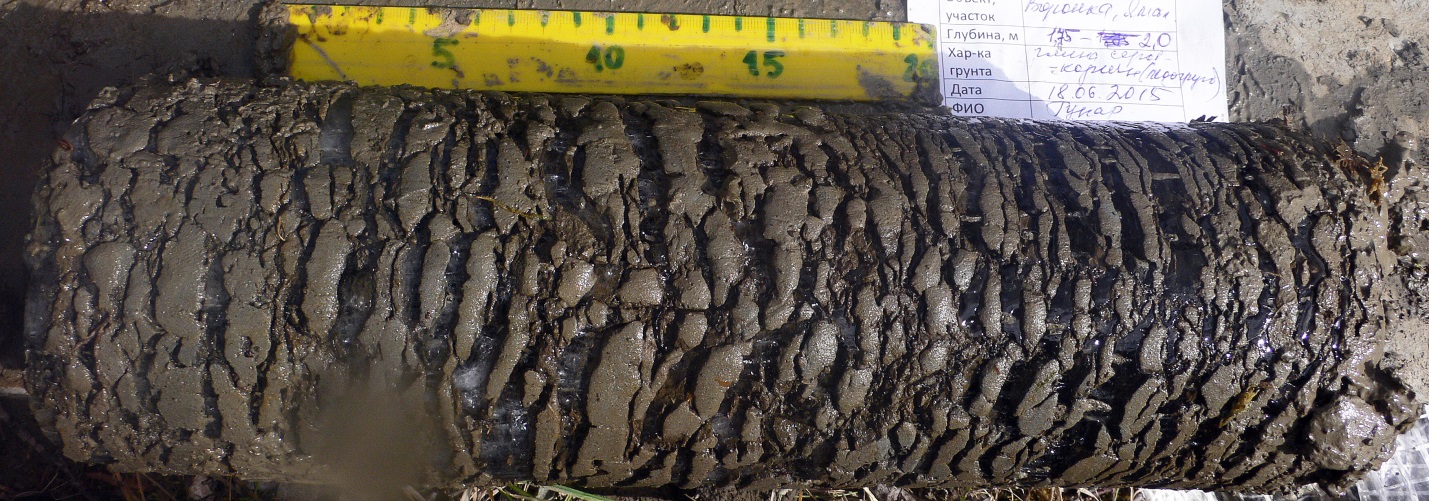


B


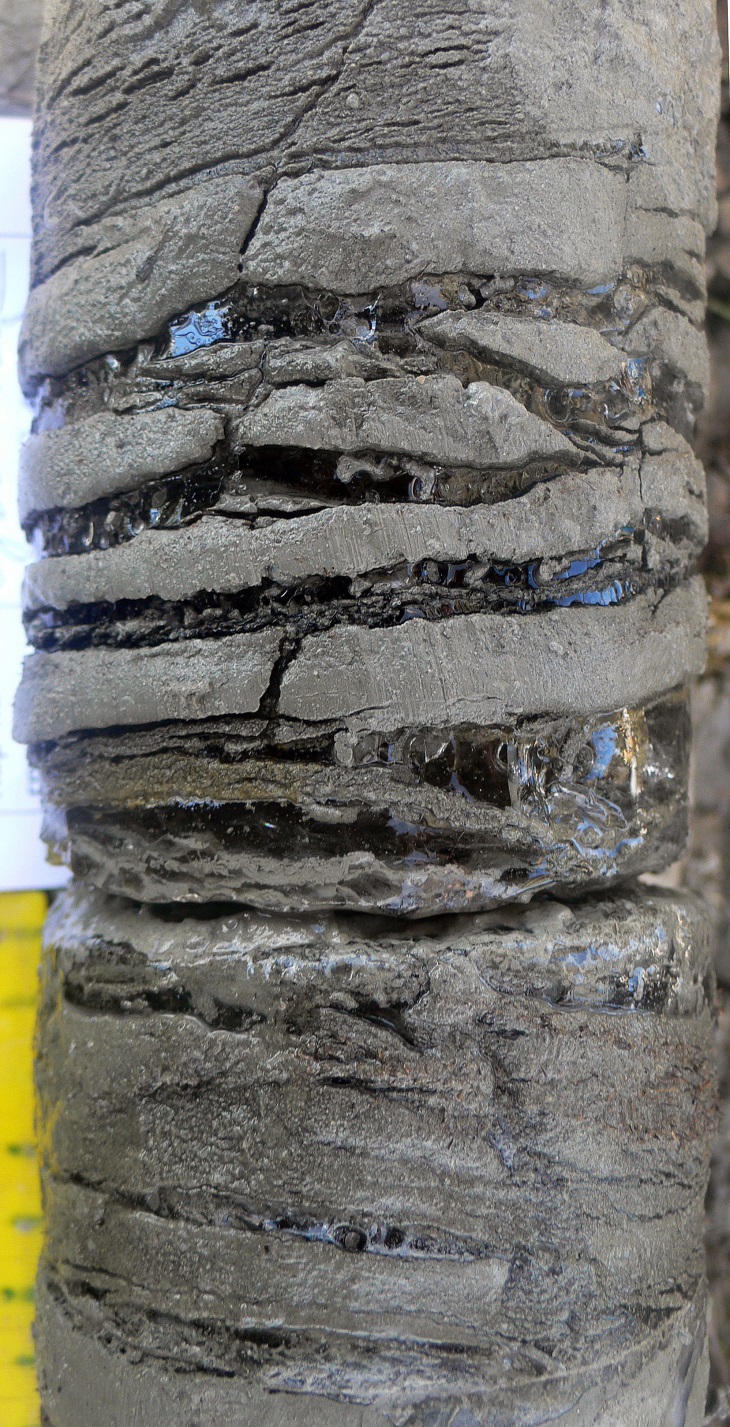


C


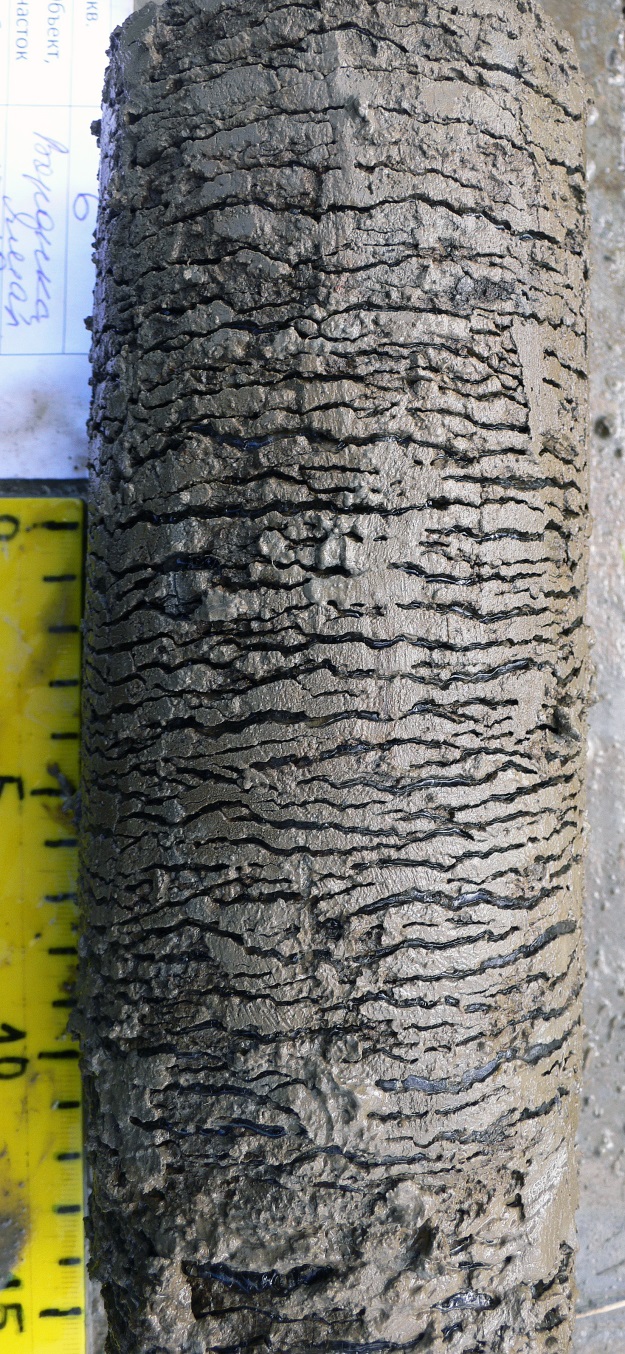


Fig. S5. Сryostrucures of talik soils.

A - Irregular reticulate cryostructure. Borehole 2, 1.75-2.0 m depth.

B - Layered cryostructure. Thick horizontal ice layers with infrequent thin vertical ice joint (junction). Borehole 2, 5.5-6.05 m depth.

C - Lenticular cryostucture. Borehole 6, 1.5-1.8 m depth. Centimeter ruler for scale.

Photograph by Vanda Khilimonyuk.

A


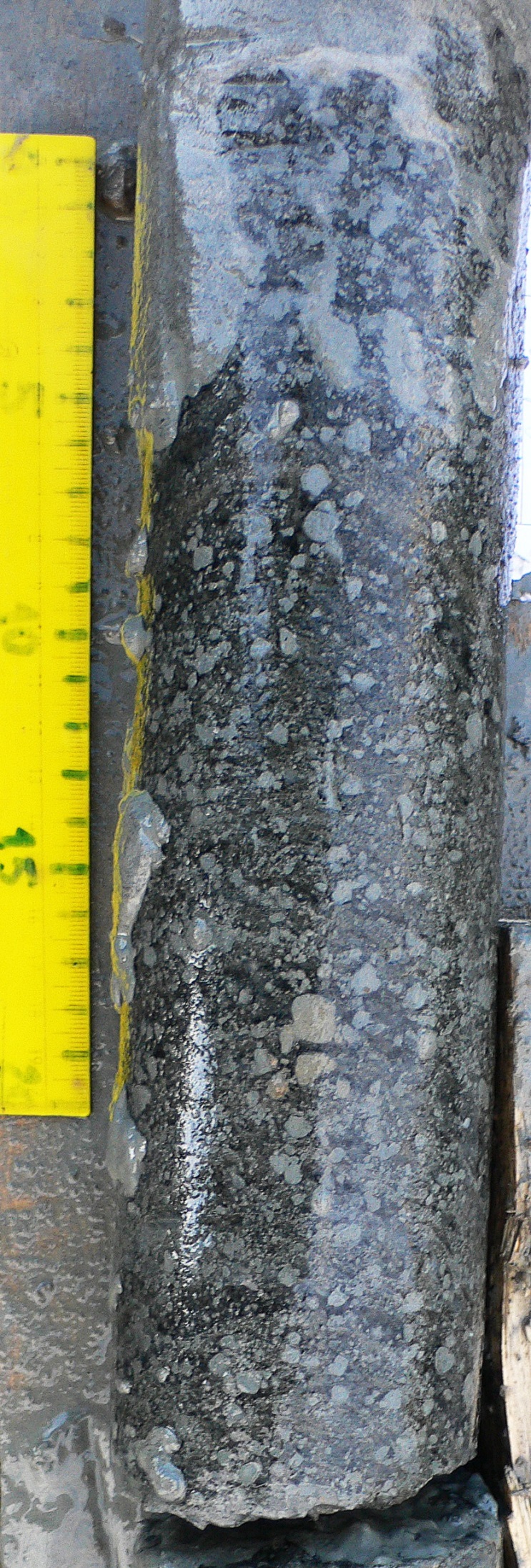


B


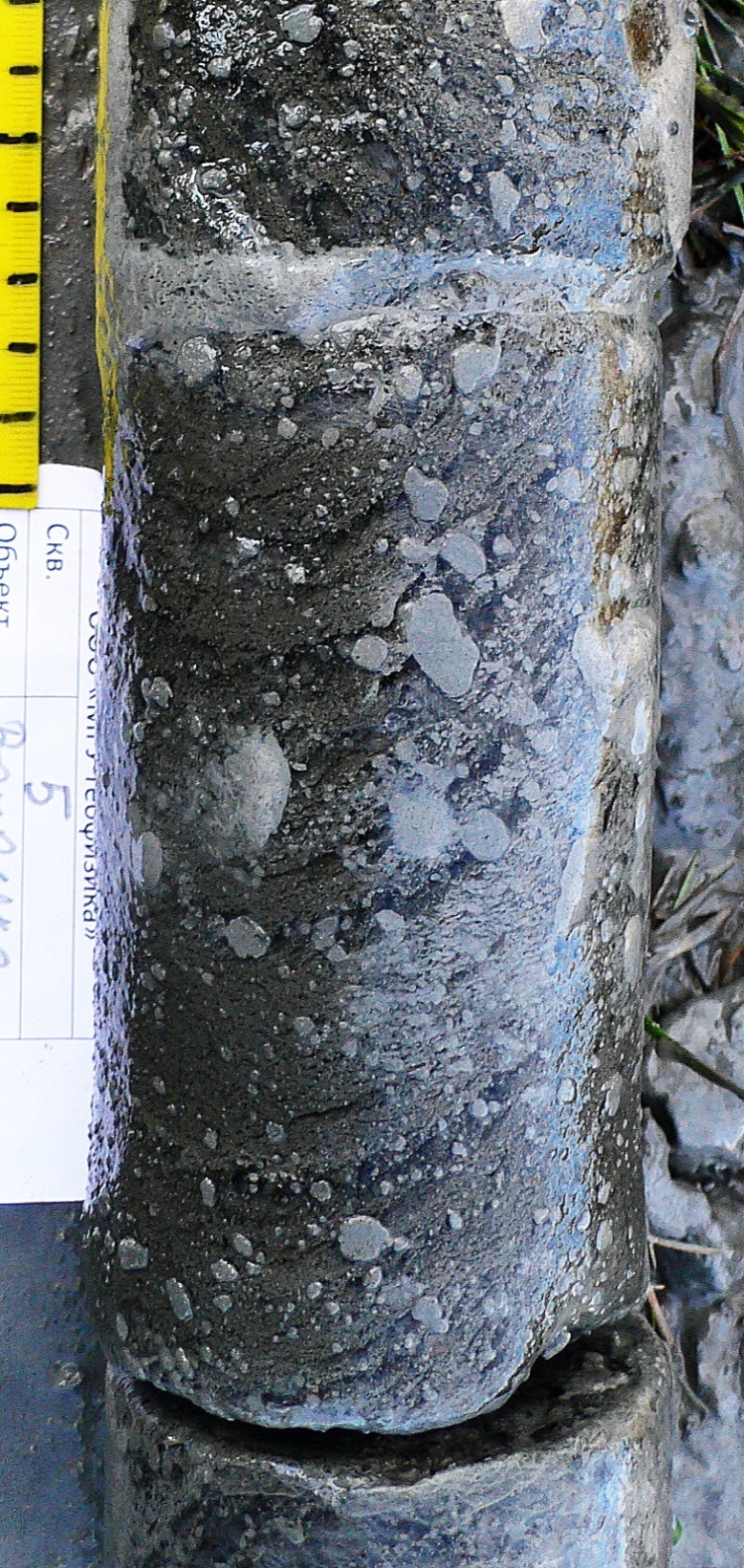


C


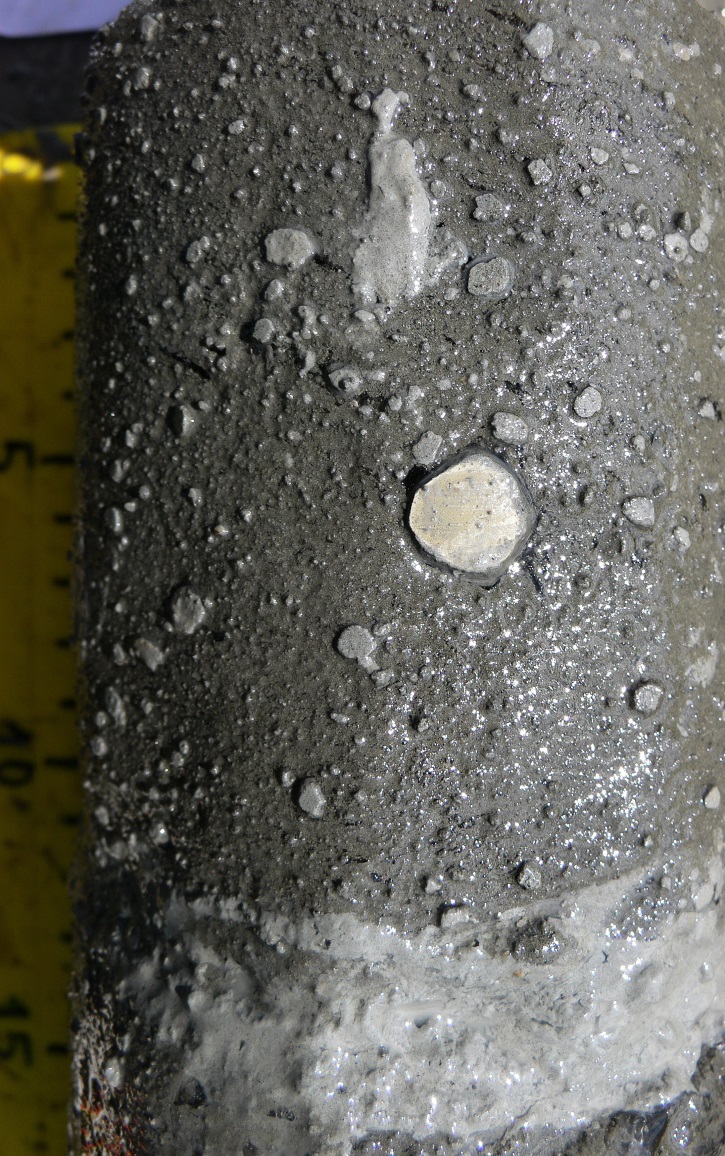


Fig. S6. Сryostrucures of talik soils.

A - Suspended cryostructure. Borehole 3, 9.0-9.6 m depth. B - Lenticular cryostructure. Borehole 5, 7.0-7.6 m depth. C - Structureless cryostructure of the sand with gravels. Borehole 6, 2.1-2.4 m depth. Centimeter ruler for scale. Photograph by Vanda Khilimonyuk.


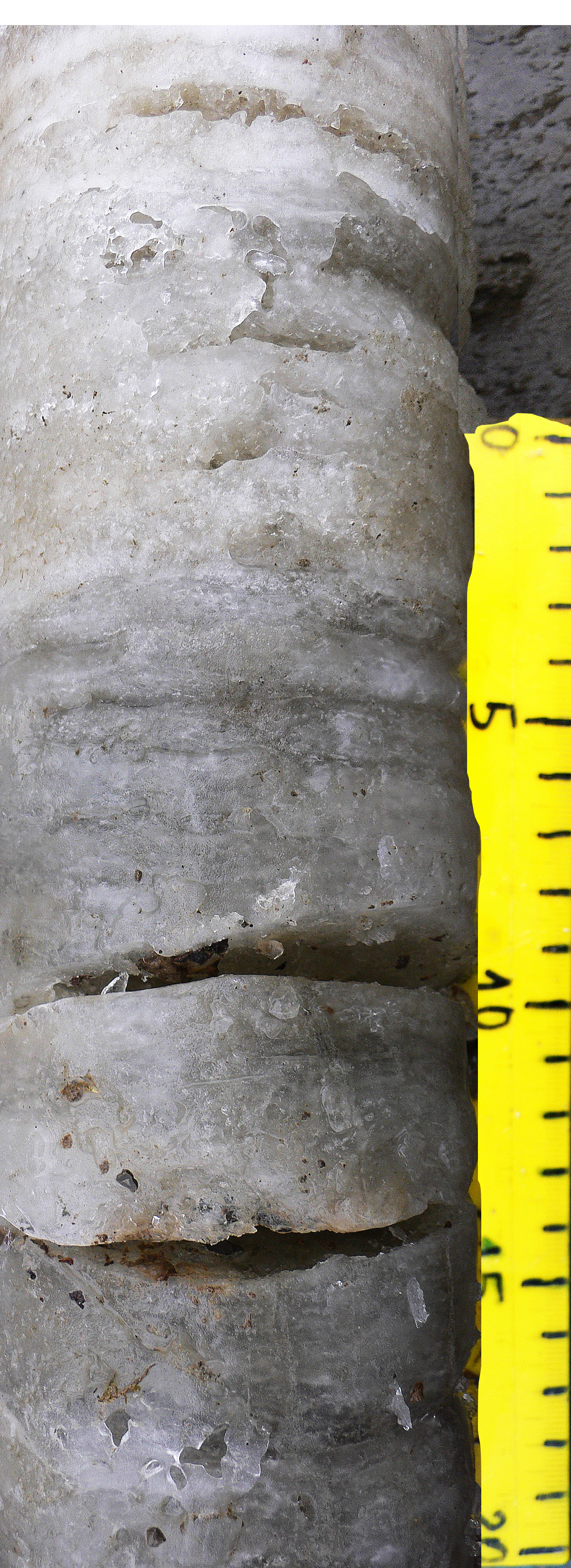


Fig. S7. Layer of pure injected “milky” ice.

Borehole 7, 5.8-6.3 m depth. Centimeter ruler for scale. Photograph by Vanda Khilimonyuk.


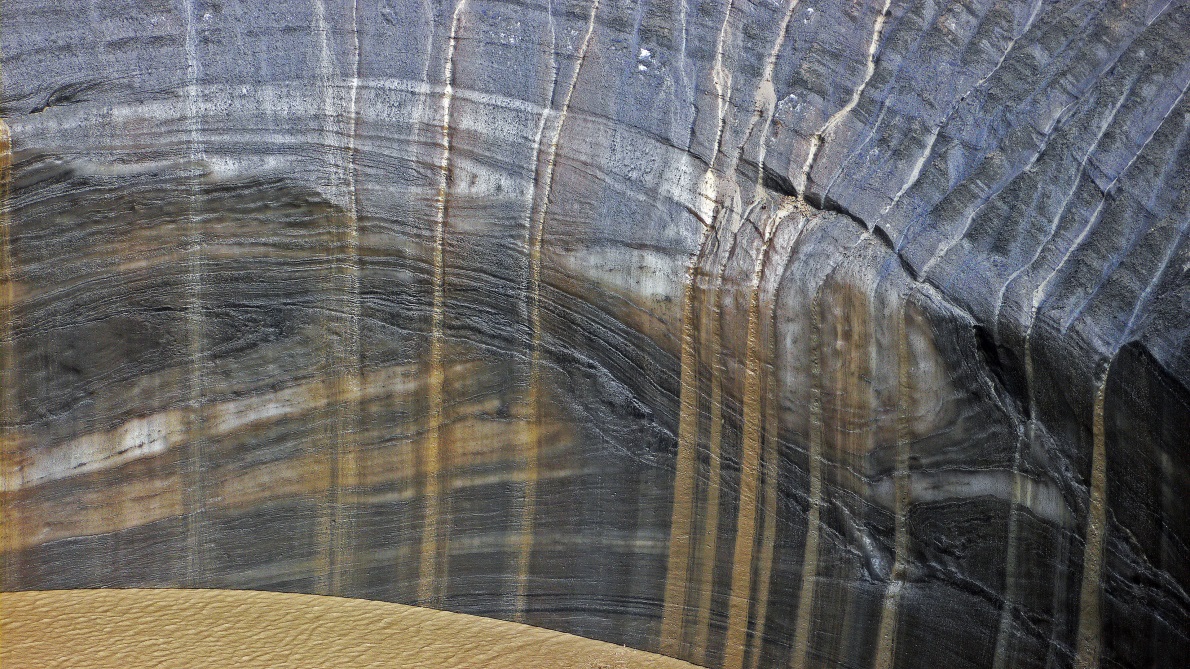


Fig. S8. Layer of injected ice in the crater.

The length covered by the photo is around 20-25 m. Photograph by Vanda Khilimonyuk.

A


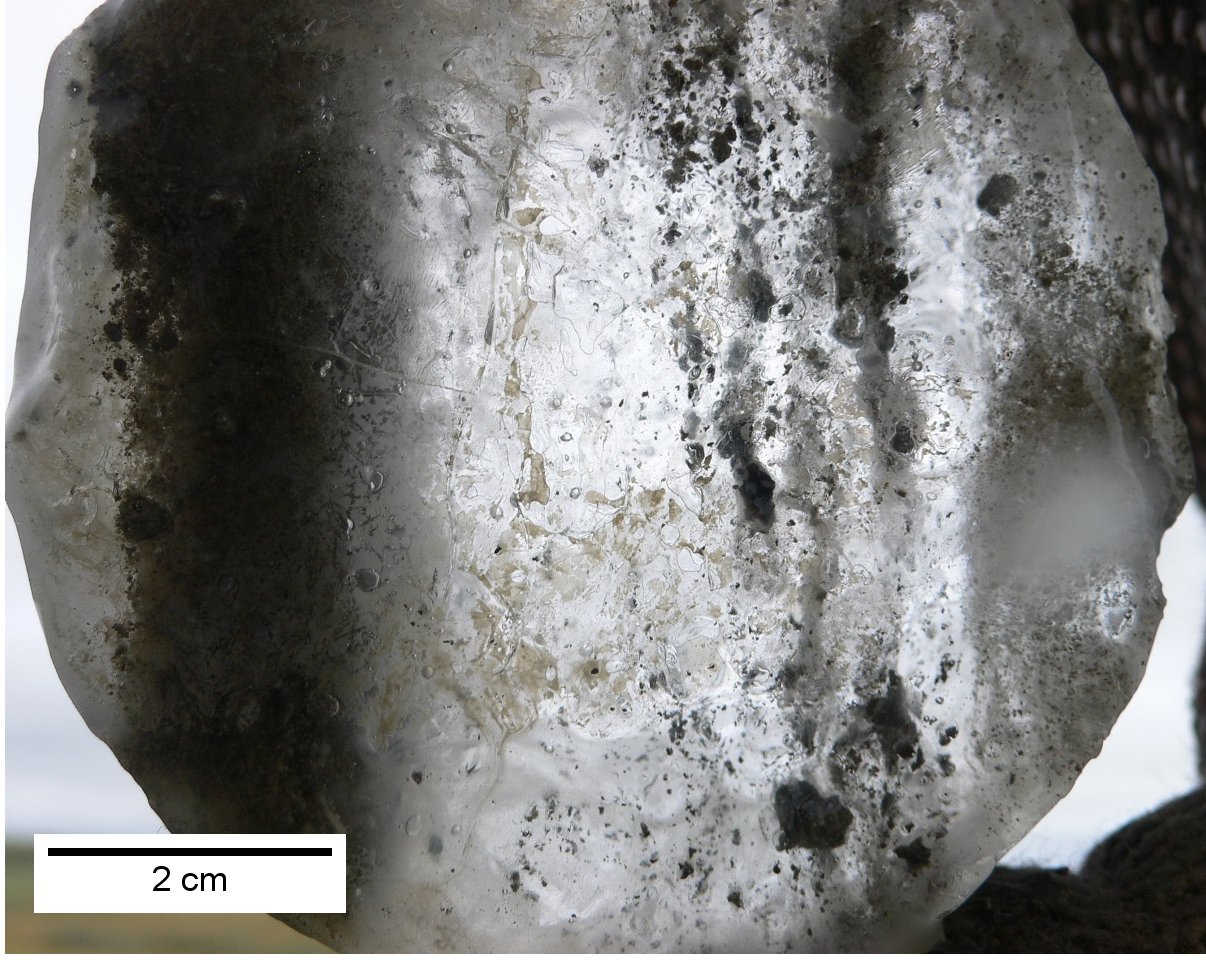


B


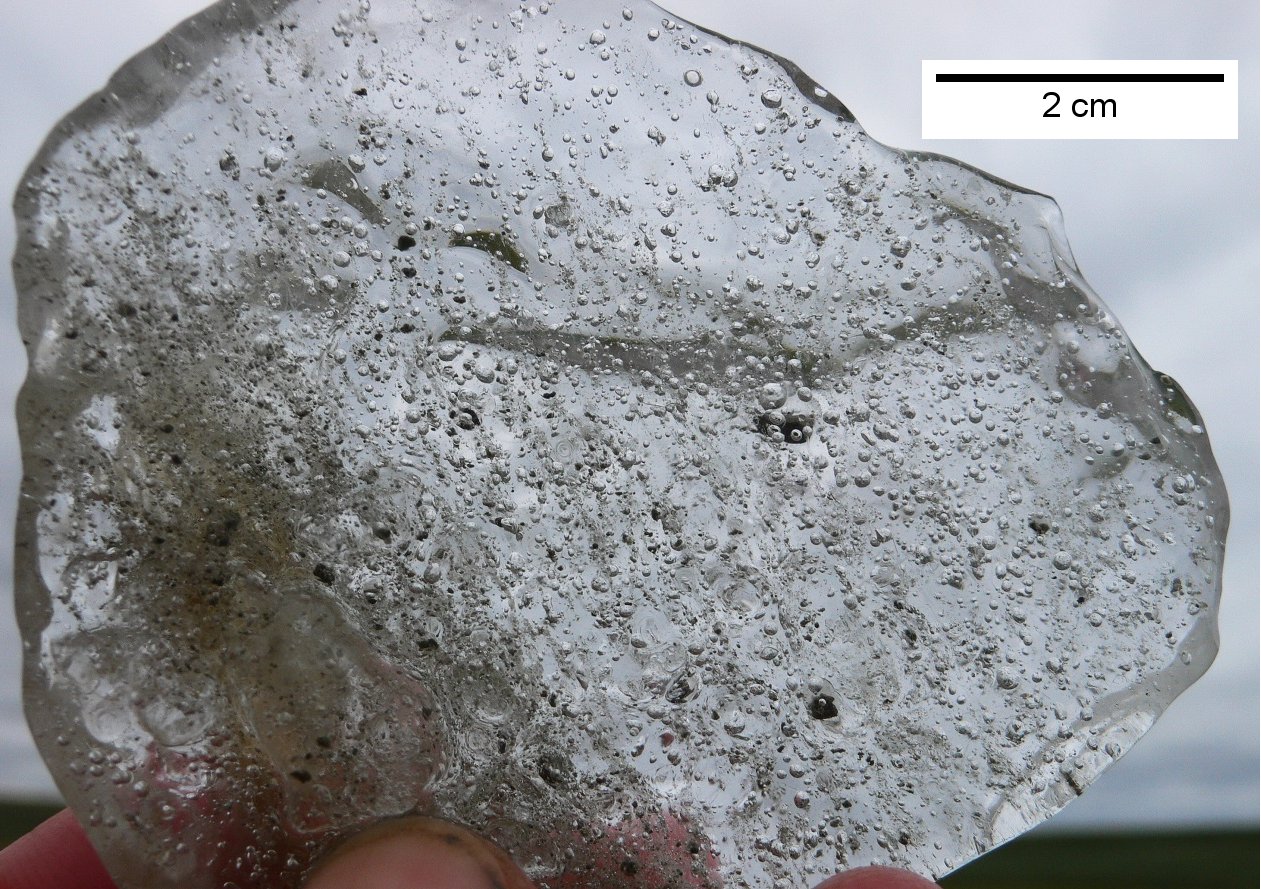


Fig. S9. Layers of pure injected ice.

A - Ground inclusions orient in vertical direction. Borehole 7, 10.5 m depth.

B - Air bubbles locate in one direction with clearly orientation. Borehole 7, 7.5 m depth. Photograph by Vanda Khilimonyuk.

A


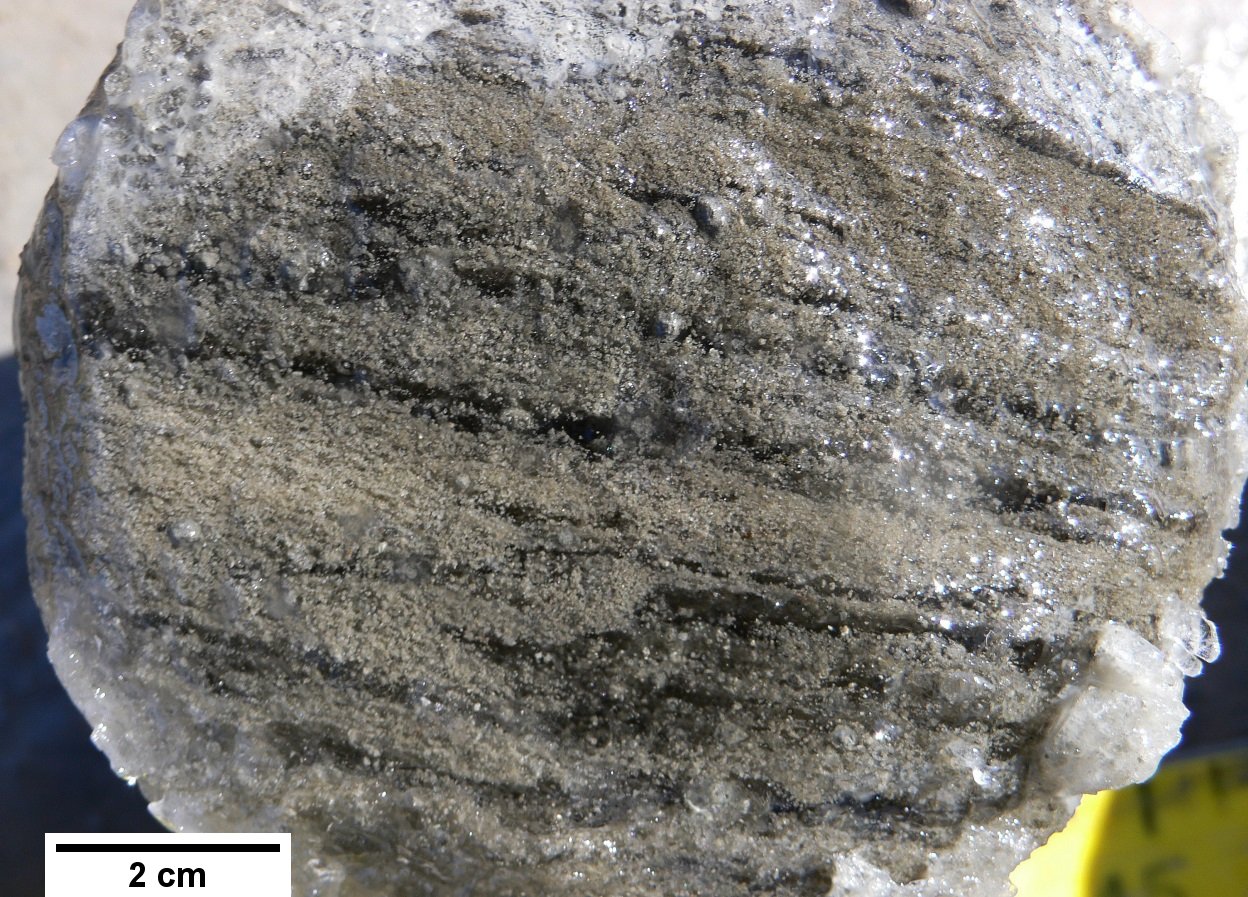


B


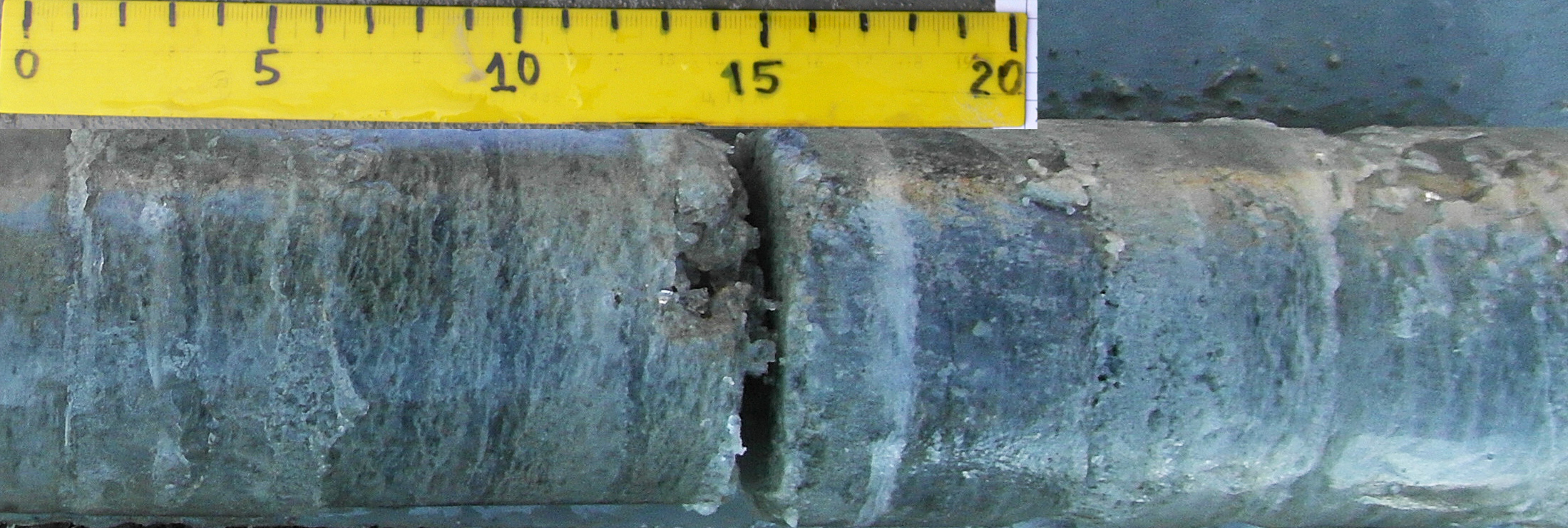


Fig. S10. Cryostructures of ice near crater.

A - Layered cryostructure. Vertical stratification. Borehole 7, 11.6-12.0 m depth.

B - Suspended cryostructure. Vertical stratification. Borehole 7, 15.5-16.0 m depth.

Photograph by Vanda Khilimonyuk.

Fig. S11. The composition of mineral components in H_2_O-free basis of ground ice(wt %).

Y, ppm

Fig. S12. The concentration of some elements in the melting ice.

Fig. S13. Gas content in ground ice

Total gas concentration is presented in vol.% at normal conditions normalized to ground ice volume. For CO_2_ and CH_4_ - vol.% in gas mixture at normal conditions.
